# Cytokine Storm: The Primary Determinant for the Pathophysiological Evolution of COVID-19 Deterioration

**DOI:** 10.3389/fimmu.2021.589095

**Published:** 2021-04-28

**Authors:** Ruirong Chen, Zhien Lan, Jujian Ye, Limin Pang, Yi Liu, Wei Wu, Xiaohuan Qin, Yang Guo, Peidong Zhang

**Affiliations:** ^1^ Department of Cardiology, Heart Center, Zhujiang Hospital, Southern Medical University/The Second School of Clinical Medicine, Southern Medical University, Guangzhou, China; ^2^ Department of Neurology, Zhujiang Hospital, Southern Medical University, Guangzhou, China

**Keywords:** cytokine storms, severe acute respiratory syndrome coronavirus 2 (SARS-CoV-2), acute respiratory distress syndrome (ARDS), multi-organ dysfunction syndrome (MODS), coronavirus disease 2019 (COVID-19)

## Abstract

The coronavirus disease 2019 (COVID-19) pandemic caused by the novel severe acute respiratory syndrome coronavirus 2 (SARS-CoV-2) is an ongoing major threat to global health and has posed significant challenges for the treatment of severely ill COVID-19 patients. Several studies have reported that cytokine storms are an important cause of disease deterioration and death in COVID-19 patients. Consequently, it is important to understand the specific pathophysiological processes underlying how cytokine storms promote the deterioration of COVID-19. Here, we outline the pathophysiological processes through which cytokine storms contribute to the deterioration of SARS-CoV-2 infection and describe the interaction between SARS-CoV-2 and the immune system, as well as the pathophysiology of immune response dysfunction that leads to acute respiratory distress syndrome (ARDS), multi-organ dysfunction syndrome (MODS), and coagulation impairment. Treatments based on inhibiting cytokine storm-induced deterioration and occurrence are also described.

## Introduction

SARS-CoV-2 is the pathogen responsible for the COVID-19 global pandemic. As it is a novel and strongly infectious coronavirus, most people lack immunity to it. Studies have shown that the cytokine storm is closely associated with disease exacerbation and even death in COVID-19 patients (1, 2). When the virus invades the body, it can cause an imbalance in the immune system that may result in a cytokine storm. COVID-19 patients deteriorate over a short period, leading to ARDS and coagulation disorders, and eventually also multiple organ failure. Understanding the pathophysiological processes involved in COVID-19 progression due to cytokine storms is extremely important, as each key link is expected to be a potential target for immune intervention against COVID-19 and will also help in the development of therapeutic drugs to prevent disease deterioration.

## The Cytokine Storm and Covid-19

COVID-19 patients can develop a fever and dry cough, and some may also experience dyspnea, muscle and/or joint pain, headache, dizziness, diarrhea, nausea, and hemoptysis (3, 4). In severe cases of COVID-19, organs can be damaged to varying degrees and even result in MODS. COVID-19 can be complicated by ARDS, acute heart injury (1), acute kidney injury, septic shock, liver injury (4), and pancreatic tissue injury (5), among others. The effect of COVID-19 on the cardiovascular system is more severe in patients with elevated levels of inflammatory factors such as interleukin (IL)-6. In particular, a burst of inflammatory factors can result in diffuse microvascular thrombosis, leading to myocarditis, heart failure, arrhythmia, acute coronary syndrome, rapid deterioration, and even sudden death (6).

The lungs are the organ most commonly affected in COVID-19 patients and display pathological features very similar to those of SARS or Middle East respiratory syndrome (MERS) coronavirus infection (7). Under macroscopic observation, patients with severe COVID-19 exhibit a large number of sticky secretions emanating from the alveoli, as well as some fibrous cords. White foamy mucus can be seen in the airway cavity, and sticky gelatinous mucus is observed in the bronchial cavity. Pathological examination shows significant desquamation of lung cells and alveolar hyaline membrane formation, suggestive of ARDS (8). In patients who die from COVID-19-related or flu-related respiratory failure, the histological pattern of the lungs manifests as a diffuse alveolar injury with perivascular T-cell infiltration. Additionally, the incidence of microthrombus and the number of new blood vessels are both higher in COVID-19 patients than in influenza patients (9). On admission, the typical chest CT findings of patients with severe COVID-19 are bilateral, multiple, lobular, and subsegmental consolidation, and even whole-lung consolidation with “white lung” appearance (10, 11). Laboratory tests show that the number of lymphocytes is significantly reduced in COVID-19 patients; in particular, the levels of CD4^+^ T and CD8^+^ T cells in patients with severe disease show a significant and progressive decline (11, 12). Concurrently, the plasma levels of IL-1β, Interleukin-1 receptor antagonist (IL1RA), IL-2, IL-7/8/9/10, basic fibroblast growth factor (BFGF), granulocyte colony-stimulating factor (GCSF), granulocyte-macrophage colony-stimulating factor (GM-CSF), interferon gamma (IFN-*γ*), interferon gamma-induced protein 10 (IP-10), monocyte chemotactic protein 1 (MCP-1), macrophage inflammatory protein 1-alpha (MIP-1-*α*), macrophage inflammatory protein 1-beta (MIP-1-*β*), platelet-derived growth factor (PDGF), tumor necrosis factor (TNF), and vascular endothelial growth factor (VEGF) are significantly increased in patients with severe COVID-19, among which IL-6 was significantly increased (1). The main cell sources, target cells, and primary immunobiological effects of the above-mentioned cytokines are shown in **Table 1**.

**Table 1:** Cytokines in COVID-19.

Cytokine	Main cellular source	Target cell	Main immunobiological effect
IL-1α	Monocytes Macrophages Neutrophils	CD4+ T cells B cells Monocytes Macrophages NK cells Neutrophils	– Activates CD4+ T cells, NK cells, neutrophils, macrophages, monocytes, and B cells – Pyrogenic
IL-1β	Monocytes Macrophages Neutrophils	CD4+ T cells B cells Monocytes Macrophages NK cells Neutrophils	– Activates CD4+ T cells, NK cells, neutrophils, macrophages, monocytes, and B cells – Pyrogenic
IL-2	CD4+ T cells B cells NK cells Monocytes Macrophages	T cells B cells Macrophages	– Stimulates T cells and B cells to grow and produce cytotoxic factors – Promotes antibody secretion – Activates macrophages
IL-7	Marrow stroma cells	Precursor B cells Thymic cells Macrophages	– Stimulates the division of precursor B cells – Promotes the maturation of double-negative thymocytes
IL-9	Th cells	Th cells Mastocytes	– Maintains long-term Th cell growth – Promotes mast cell growth and activity
IL-8	Monocytes Macrophages Neutrophils Lymphocytes	Neutrophils T lymphocytes Basophil granulocytes	– Enhances the chemotactic effects of neutrophils, T lymphocytes, basophils
IL-10	Th2 cells Monocytes Macrophages Activation of B cells	Th1 cells Activated T cells B cells NK cells Monocytes Macrophages	– Inhibits cellular immunity to promote humoral immunity
BFGF	Macrophages Endothelial cells Smooth muscle cells	Endothelial cells Smooth muscle cells	– Promotes the proliferation of endothelial cells and smooth muscle cells
GCSF	Monocytes Macrophages	Neutrophils Monocytes Macrophages	– Stimulates granulocyte, monocyte, and macrophage maturation and release into the peripheral blood
GM-CSF	B cells Macrophages T cells	Neutrophils Monocytes Macrophages	– Stimulates granulocyte, monocyte, and macrophage maturation and release into the peripheral blood – Enhances non-specific immunity
PDGF	Platelet cells Monocytes Macrophages	Macrophages Fibroblasts Neutrophils Vascular smooth muscle cells	– Enhances non-specific immune cells and chemotaxis – Shrinks blood vessels – Stimulates the proliferation of vascular smooth muscle cells and fibroblasts – Promotes prostaglandin production
VEGF	Endothelial cells	Endothelial cells Monocytes	– Promotes the growth of vascular endothelial cells – Increases capillary permeability – Enhances monocyte chemotaxis
IFN-*γ*	Monocytes Macrophages Lymphocytes	Macrophages Neutrophils Monocytes Vascular endothelial cells	– Enhances cytotoxic effects -Injures the vascular endothelial cells – Chemotaxis and activates neutrophils, monocytes, and macrophages
IP-10	Monocytes Macrophages T cells	Monocytes cells Lymphocytes NK cells	– Chemotactic monocytes, lymphocytes, and NK cells
MCP-1	Macrophages Fibroblasts Lymphocytes Endothelial cells	Mononuclear cells Lymphocytes Basophils	– Promotes chemotaxis and activates monocytes, lymphocytes, and basophils
MIP-1-α	Macrophages Activated T lymphocytes Neutrophils Monocytes Fibroblasts Mastocytes	CD4+ T cells CD8+ T cells Monocytes Eosinophils Mastocytes Basophils	– Promotes chemotaxis and activates CD4+ T cells, CD8+ T cells, monocytes, eosinophils, mast cells, basophils, and neutrophils – Induces eosinophils to release ECP – Stimulates mast cells and eosinophils to release histamine
MIP-1-*β*	Monocytes Macrophages Neutrophils	CD4+ T cells CD8+ T cells Monocytes Neutrophils	– Chemotactic CD4+T cells, CD8+T cells, monocytes, neutrophils
TNF	Monocytes Macrophages	Endothelial cells Lymphocytes NK cells Neutrophils	– Enhances cytotoxic effects – Injures the endothelial cells – Increases phagocytosis of neutrophils

L-1α, interleukin 1 alpha; IL-1β, Interleukin 1 beta; IL-2, interleukin 2; IL-7, interleukin 7; IL-8, Interleukin 8; IL-9, interleukin 9; IL-10, interleukin 10; BFGF, basic fibroblast growth factor; GM-CSF, granulocyte-macrophage colony-stimulating factor; GCSF, granulocyte colony-stimulating factor; PDGF, platelet-derived growth factor; VEGF, vascular endothelial growth factor; IFN-*γ*, interferon gamma; IP-10, interferon-inducible protein 10; MCP-1, monocyte chemotactic protein 1; MIP-1-*α*, macrophage inflammatory protein 1-alpha; MIP-1-*β*, macrophage inflammatory protein 1-beta; TNF, tumor necrosis factor.

The above-mentioned clinical characteristics of COVID-19 patients suggest that the accumulation and exudation of inflammatory substances caused by the cytokine storm destroy tissues and organs throughout the body, leading to multi-organ failure and acute respiratory distress (13), which is an important cause of death in patients with severe and critical cases of COVID-19. The determinants of the severity of COVID-19 disease deterioration are closely related mainly to the cytokine storm and a decrease in lymphocytosis, which are key early warning factors for the transition to severe disease (12).

## Activation and Intensification of the Cytokine Storm in Covid-19 Patients

Adaptive immunity is suppressed in severe COVID-19 patients, leading to delayed clearance of the virus and hyperactivation of the innate immune response. However, the innate immune response makes it difficult to fight against the virus. The lack of negative feedback, as well as positive feedback aggravation, promotes the overproduction of various inflammatory factors, leading to an increase in the number of active immune cells at the sites of inflammation and, consequently, a cytokine storm (6).

Angiotensin-converting enzyme 2 (ACE2) is a key receptor for coronavirus invasion. Studies have shown that the binding of SARS-CoV-2 to host cell membrane receptors and subsequent fusion with the cell membrane are mainly mediated by the spike (S) protein of the virus, which is closely associated with its pathogenicity (14, 15). Furthermore, transmembrane serine protease 2 (TMPRSS2) is required for the virus to enter host cells, while ACE2 is considered to be the limiting factor for the initial SARS-CoV-2 infection (16). ACE2 and TMPRSS2 are reported to be co-expressed mainly in type II alveolar epithelial cells, nasal epithelial cells, cornea, esophagus, ileum, colon, gallbladder, and common duct (16, 17). However, because most COVID-19 patients display lung manifestations, while relatively fewer present with abdominal pain, abdominal distention, diarrhea, and other digestive system symptoms, this suggests that the main target organ of SARS-CoV-2 is likely to be the lung (18).

**Figure 1:**
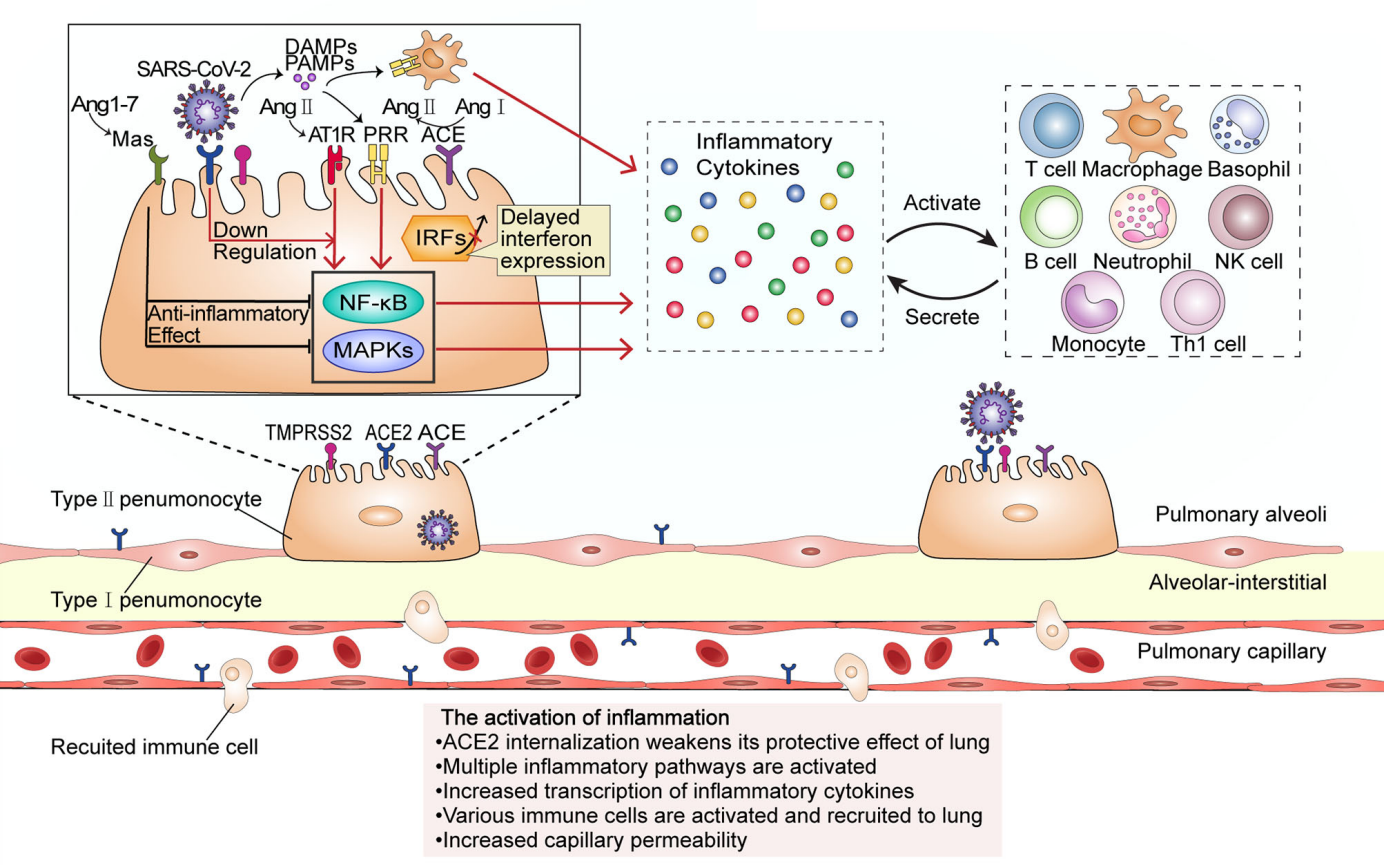
The activation of inflammation during SARS-CoV-2 infection. The virus enters the host cell by interacting with the ACE2 receptor and the cellular serine protease TMPRSS2 through its spike protein. ACE2 internalization weakens the anti-inflammatory ACE2/Ang-(17)/MAS receptor axis maintained by ACE2. At low SARS-CoV-2 viral load, cells do not initiate the interferon response; however, upregulation of Ang II stimulates its receptor AT1R and promotes the transcription of several inflammatory cytokine-related genes through proinflammatory pathways such as the NF-*κ*B and MAPK signaling pathways. Proinflammatory cytokines activate and recruit a variety of immune cells to migrate to the site of infection, and the activated immune cells will secrete more inflammatory cytokines. In this stage, the inflammation leads to an increase in capillary permeability and consequent liquid exudation. PRR, pattern recognition receptor; PAMPs, Pathogen-associated molecular patterns; DAMPs, damage-associated molecular patterns.

SARS-CoV-2 infects alveolar epithelial cells by recognizing the ACE2 receptor. Following cell invasion, the virus replicates in large quantities, which activates immune cells and the release of a large number of inflammatory cytokines, leading to a cytokine storm (**Figure 1**). The internalization and exfoliation of ACE2 reduced the expression of ACE2 on the cell membrane but did not affect the level of ACE, resulting in an imbalance of ACE2 and ACE in the lung (17, 19). ACE2 has a lung-protective effect (20). Reduced levels of ACE2 weaken its lung-protective effect, which may result in alveolar epithelial cell necrosis and aggravation of lung inflammation (19). Increased ACE expression upregulates the level of angiotensin II (Ang II) and further hyperactivates the pulmonary angiotensin II type-1 receptor (AT1R), thereby increasing pulmonary capillary permeability and leading to pulmonary edema and aggravation of lung damage. Ang II is able to bind to the AT1R to stimulate the Janus Kinase (JAK)/Signal transducers and activators of transcription (STAT) pathway and facilitates the production of downstream IL-6, which in turn triggers the JAK/STAT pathway through positive feedback to release more cytokines such as IL-6 and IFN (21). Ang II can also combine with nuclear factor kappa-B (NF-*κ*B) and promote the transcription and production of inflammatory cytokines such as IFN-*γ*, IL-6, GM-CSF, TNF-*β*, and TNF-α (22). The plasma level of Ang II was reported to be significantly increased in SARS-CoV-2-infected patients and shows a linear correlation with viral load and lung injury (3). Additionally, Ang-(1–7) which is produced by ACE2 degradation of Ang II decreases, and the anti-inflammatory effect of the PIP3/Akt and ERK signaling pathway regulated by the MAS receptor specifically binding with Ang-(1–7) should be weakened (23). This promotes the cytokine storm, and accelerates the deterioration of patients to ARDS and multiple organ dysfunction, eventually leading to the death of patients (3).

Damage-associated molecular patterns (DAMPs) produced by virus-infected epithelial cells and pathogen-associated molecular patterns (PAMPs) of the virus itself can combine with pattern recognition receptors (PRRs) expressed in pulmonary epithelial cells (24), macrophages, and dendritic cells, and then activate NF-*κ*B and several mitogen-activated protein kinases (MAPKs) such as ERK1/2, p38, JNK, ERK5, triggering a series of cascades and release of chemokines. Normally, NF-*κ*B inhibited protein (I*κ*BS) is transported in the cytoplasm. SARS-CoV-2 promotes nuclear translocation of NF-*κ*B by inducing phosphorylation, ubiquitination, and degradation of I*κ*B through proteasomes. In the nucleus, NF-*κ*B induces the transcription of several genes, which encode TNF-α, IL-6, and other cytokines and enhance the occurrence of cytokine storm (25). MCP-1, MIP-1-*α*, MIP-1-*β*, IP-10 chemotactic lymphocytes, macrophages, neutrophils, natural killer (NK) cells, basophils, and other immune cells gather at the site of injury; the interleukins IL-1*α*, IL-1β, IL-2, IL-8, and IL-10, among others, activate and strengthen lymphocytes, macrophages, neutrophils, NK cells, basophils, and other immune cells; IFN-*γ* and TNF enhance cytotoxicity; the release of the colony-stimulating factors GCSF and GM-CSF stimulates granulocyte, monocyte, and macrophage maturation and release into the blood; growth factors such as PDGF, VEGF, and basic FGF stimulate the proliferation of endothelial cells, smooth muscle cells, and fibroblasts (26). The activated immune cells generate new cytokines, recruit and activate more inflammatory cells, and release more cytokines, and this cascade amplification will finally lead to a cytokine storm. IL-6 is a key inducer of the cytokine storm, a key factor in disease deterioration (27, 28). Cell and animal experiments, as well as results from human patients, have all shown that interferon (IFN) expression is reduced following SARS-CoV-2 infection (29). The levels of type I and III interferons are exceedingly low, and interferon expression is only activated at a high multiplicity of infection. IFN and other downstream molecules (including proinflammatory cytokines) controlled by IFN have a variety of functions such as direct inhibition of viral replication and recruitment and activation of various immune cells and are the first line of defense against viral infection (30, 31). In contrast, this virus can induce elevated expression levels of IL-6 and IL-1RA, as well as other proinflammatory factors. This indicates that the host innate antiviral immunity is dysregulated and is likely to lead to a cytokine storm outbreak (32, 33).

Activated CD4^+^ T cells proliferate and differentiate into T-helper 1 (Th1) cells and produce GM-CSF, IL-6, and other factors. GM-CSF further activates CD14^+^ and CD16^+^ inflammatory monocytes and activates and recruits macrophages, which produce more IL-6 and other inflammatory factors, thereby promoting cytokine storm development.

The first autopsy performed on a COVID-19 patient showed that a large number of monocytes (possibly T cells) had accumulated in the lung, and that the activity of T cells in the peripheral blood was low (8). Furthermore, the lymphocyte count and peripheral blood T-cell level have been reported to be low in COVID-19 patients (11, 34). These findings indicate that T cells are attracted from the blood to the infected site to control the viral infection.

**Figure 2:**
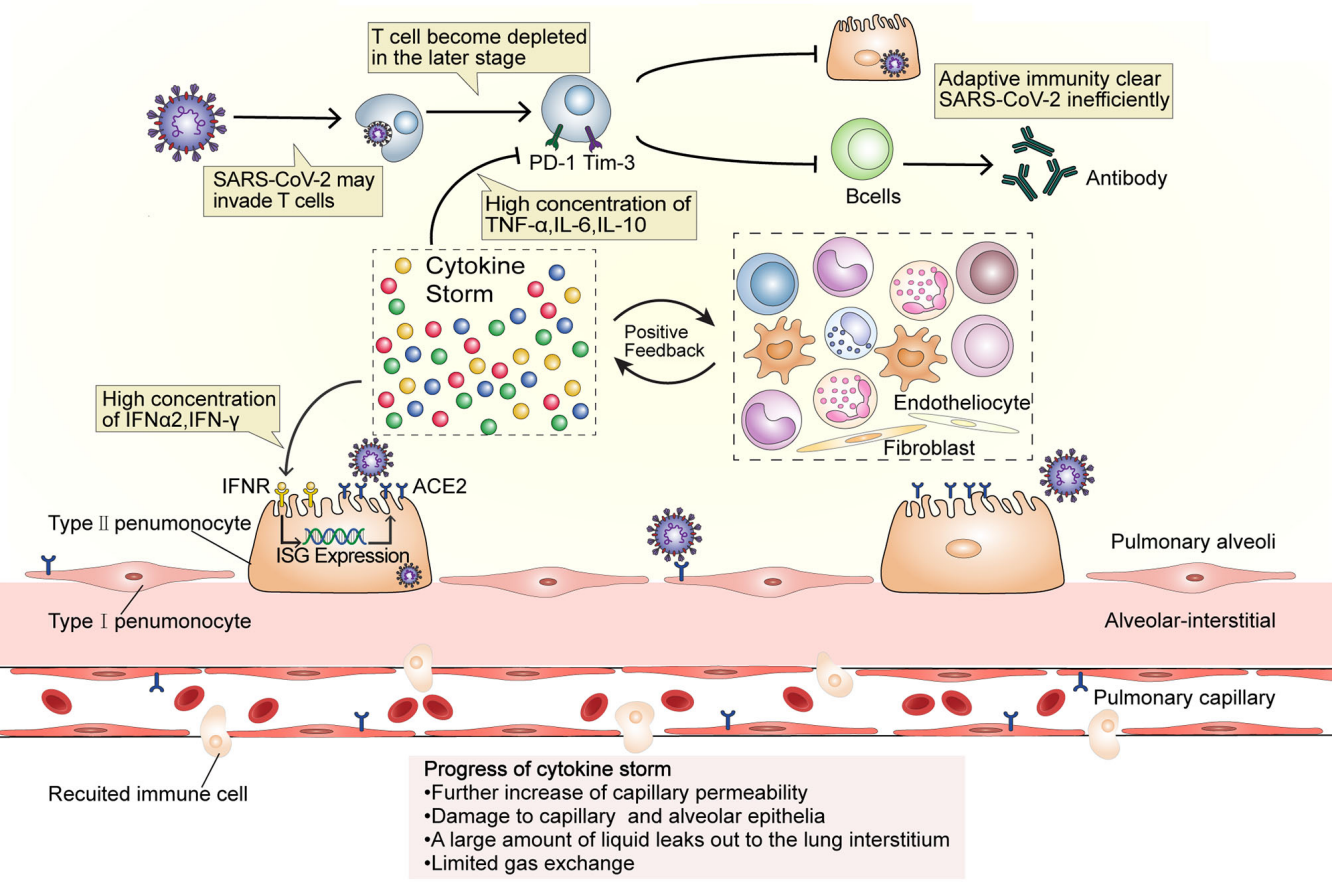
A dysfunctional immune response induces cytokine storms. SARS-CoV-2 may invade T cells that are attracted to the site of infection and replicates inside them. At the later stage of COVID-19, T cells become depleted and the expression of PD-1 and Tim-3 increases, while the high IL-10, TNF, and IL-6 concentration affect T cell survival or proliferation. The number of B cells also decreases in patients with severe disease. The dysfunction of adaptive immunity results in the magnification of innate immunity and establishes an inflammatory feedback loop. High concentrations of IFN-α2 and IFN-*γ* may upregulate the expression of ACE2 during the cytokine storm. Additionally, as ACE2 is an ISG, its internalization will further induce ACE2 expression and eventually provide more receptors for virus invasion and aggravate the infection, thus intensify the inflammation. In this stage, capillary permeability is further increased and the capillary and alveolar epithelia become damaged, causing the leakage of a large amount of protein-rich liquid into the lung interstitium and limiting gas exchange. ISG, interferon-stimulating gene.

The numbers of CD4^+^ and CD8^+^ T cells in peripheral blood significantly reduce in severe COVID-19 (35, 36). T cells may be infected by SARS-CoV-2, which is similar to the occurrence in Middle East respiratory syndrome (MERS) cases (**Figure 2**). The MERS coronavirus can directly infect human primary T lymphocytes and induce T-cell apoptosis through extrinsic and intrinsic apoptotic pathways, but it cannot replicate in T lymphocytes (37). The numbers of Th17 CD4^+^ T cells, which play a proinflammatory role, were increased and CD8^+^ T cells were highly cytotoxic (1). This suggests that a decrease in the levels of CD4^+^ and CD8^+^ T cells leads to dysregulated innate and adaptive immune responses. The impaired immune response delays virus clearance, which stimulates non-specific immune cells, and leads to the release of more proinflammatory factors (6). The high concentrations of TNF, IL-6, and IL-10 during the cytokine storm exert a negative regulatory effect on T-cell survival and proliferation. The number of T cells is negatively correlated with the concentrations of IL-6, IL-10, and TNF. Furthermore, the expression of PD-1 and Tim-3 in T cells is upregulated from initial infection to disease onset, further indicating T-cell failure (35, 36) and leading to a vicious cycle. B cell immune responses and follicular helper T-cell responses occur simultaneously in COVID-19 patients (38). In addition to producing antibodies, activated B cells also secrete IL-1, IL-6, IL-8, TNF, lymphotoxin-alpha (LT-*α*), G-CSF, GM-CSF, M-CSF, IL-7, and other cytokines, which can aggravate the cytokine storm (39). The number of B cells in patients with severe disease is significantly reduced (40), meaning that not enough antibodies will be produced. Consequently, viral replication will not be inhibited, and virus infection will spread throughout the body, which will lead to a further imbalance in the immune response and the release of more proinflammatory cytokines by non-specific immune cells. Furthermore, IFNs induce cells to express a variety of proteins. The genes that express these proteins, such as ACE2, are called interferon-stimulating genes (ISGs). SARS-CoV-2 can exploit species-specific interferon-driven upregulation of ACE2. The increased expression of ACE2 increases the number of receptors available to the virus for host invasion, which inhibits the lung-protective role of ACE2 (20). Increased IFN levels during the cytokine storm have been proposed to lead to the deterioration of COVID-19 patients, further aggravating the cytokine storm.

## The Cytokine Storm Leads to the Deterioration of Covid-19 Patients

**Figure 3:**
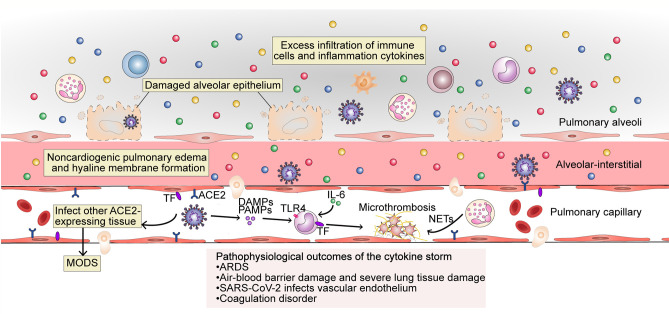
The pathophysiological outcome of the cytokine storm in SARS-CoV-2 infection. The excess filtration of immune cells and accumulation of cytokines at infected sites results in acute respiratory distress syndrome (ARDS); non-cardiogenic pulmonary edema and hyaline membrane formation; severe lung tissue damage; and destruction of the blood–blood barrier. SARS-CoV-2 can infect vascular endothelial cells that also express ACE2 and enter the blood circulation to infect other ACE2-expressing tissues. The occurrence of an inflammatory storm in other organs may lead to MODS. PAMPs, DAMPs and cytokines trigger monocyte activation and induce the membrane expression of TF on monocytes and endothelial cells. Endothelial damage can also expose TF. Under the action of cytokines (mainly IL-6), endothelial cells recruit TF-expressing inflammatory monocytes, while TF can also promote the conversion of prothrombin to thrombin, forming a fibrin-based blood clot. Recruited by activated endothelial cells, neutrophils release NETs, which activate the contact activation pathway of coagulation and platelets, thereby amplifying blood clotting. MODS, multi-organ dysfunction syndrome; TF, tissue factor; TLR, Toll-like receptor; NETs, neutrophil extracellular traps.

The cytokine storm is an important factor in the deterioration of some COVID-19 patients, and leads to abnormalities such as ARDS, MODS, and coagulation defects (4, 41, 42) (**Figure 3**).

COVID-19-related ARDS is closely related to the cytokine storm. Respiratory failure due to ARDS is the leading cause of death from COVID-19 (43). A large number of immune cells resulting from the cytokine storm aggregate and are activated in the lungs. These cells then release oxygen free radicals, proteases, and inflammatory factors through a “respiratory burst,” which damages target cells. In particular, the combined effect of TNF-α and IFN-*γ* that sensitize the cells to undergo PANoptosis, inflammatory cell death involving components of pyroptosis, apoptosis, and necroptosis plays a key role in lung tissue injury (44). As a result, lung capillary endothelial cells and lung epithelial cells are damaged, lung microvascular permeability and microthrombus formation are increased, and a large amount of protein and fibrin-rich fluids are exuded into the lung stroma and alveoli, forming non-cardiac pulmonary edemas and opacities (7, 9). Lung compliance decreases, alveolar inactivity increases, and persistent hypoxia is present, which leads to decreased Na-K-ATPase activity in alveolar epithelial cells, cellular metabolism disorders, lymphatic decompensation, and further exacerbates fluid retention and hypoxia. Concurrently, oxygen-sensitive proline hydroxylases are activated, and NF-*κ*B is released, further exacerbating inflammatory responses. Inflammatory mediators can also damage the pulmonary capillary endothelium and alveolar epithelium, leading to the contraction of vascular endothelial cells, cell connection rupture, and significantly increased vascular permeability (45). Capillary leak results from inflammation driven by key inflammatory cytokines such as TNF, IL-1, IL-6, IL-8, and especially VEGF, which in the past was also called “vascular permeability factor.” Capillary leak is a major component of deteriorating lung function in COVID-19, resulting in ARDS (46). Several proinflammatory cytokines (IL-6, L-1, and CSF), chemokines (CCL2, CCL-5, IP-10, and CCL3), and reactive oxygen species have been identified as causing ARDS (47).

Besides severe lung damage, cytokine storms can also cause cardiovascular symptoms, hematologic symptoms, acute kidney injury, and multiple organ failure, and can even be life-threatening. As mentioned above, SARS-CoV-2 mainly infects cells *via* ACE2. The virus may first infect type II alveolar epithelial cells through ACE2, leading to air–blood barrier damage. The virus can then enter the blood and infect the lungs again through ACE2 that is expressed on the endothelial cells of pulmonary capillaries. On the one hand, this leads to an increased viral load in the lungs; on the other hand, the virus binds to ACE2 on the endothelial cells, which enables the endothelial cells to be pathogen-labeled, and become recognized as targets for attack by the host immune system. Additionally, as the virus circulates to all parts of the body, it can infect other organs as well that express ACE2, such as the heart, kidney, and liver, further triggering an impaired immune response, such as an imbalance of Th1 and Th2 cells. This results in a cascade amplification, whereby a great number of inflammatory factors are produced, eventually leading to MODS.

Abnormal coagulation may be the main reason for organ failure and death in patients with severe COVID-19 (42), and a sign of organ damage in sepsis, which is mainly caused by the cytokine storm (48). Microthrombi have been reported in the heart, liver, lungs, lower limbs, hands, brain, and kidneys of COVID-19 patients (49, 50). Microthrombi occur because tissue factor (TF) is expressed both in monocytes and in vascular endothelial cells through the activity of cytokines (mainly IL-6), which is thought to promote the conversion of prothrombin to thrombin, which then converts circulating fibrinogen to fibrin and the formation of fibrin-based blood clots. Neutrophils are absorbed by activated endothelial cells and release neutrophil extracellular traps (NETs), which activate the contact activation pathway of coagulation, as well as platelets, thereby amplifying blood clot formation (48). During the cytokine storm, major natural anticoagulant effectors, such as antithrombin or TF pathway inhibitors, are always inhibited, further promoting coagulation and leading to an irreversible situation. Cytokines IL-1, IL-6, TNF, signal transducer and transcriptional activator 3 (STAT3), NF-*κ*B, and lipopolysaccharides were the highest regulators of thrombotic markers in COVID-19 patients with severe-to-critical disease (51). In the absence of vascular injury, the initiation of coagulation is completely dependent on activated endothelial cells, which express TF, for the recruitment of proinflammatory monocytes (52). The cytokine storm leads to abnormal coagulation in the body, and microthrombi formation in the organs further leads to MODS.

## Treatment and Prevention of the Cytokine Storm

Several treatments are available to prevent and suppress cytokine storm development in COVID-19 patients, such as glucocorticoids, immunomodulators, cytokines antagonists, cytokine receptor antagonists, and others (**Table 2**).

**Table 2:** Therapeutic methods linked to the prevention and inhibition of the cytokine storm in COVID-19.

Therapeutic	Therapeutic drug	Effect
***Glucocorticoids***		
–	Dexamethasone	IL-6↓
–	Prednisolone	TNF, IFN-*γ*, IL-1β, IL-6, IL-17, IL-10, TGF-*β*↓
***Immunomodulators***		
–	Thalidomide	TNF, IL-1, IL-6, IL-8↓
–	Hydroxychloroquine or chloroquine	TNF, IL-6↓
–	Ulinastatin	IL-10↑, TNF, IL-6, IFN-*γ*↓
–	Statins	MYD88/NF-*κ*B proinflammatory pathway↓ ACE2↑
***Cytokine/cytokine receptor* antagonists**		
IL-1 receptor antagonists	Anakinra	IL-1↓
IL-1β antagonists	Canakinumab	IL-1β↓
IL-6 receptor antagonists	Tocilizumab Sarilumab	IL6↓ IL6↓
IL-6 antagonists	Siltuximab Clazakizumab	IL6↓ IL6↓
GM-CSF antagonists	Lenzilumab	granulocytes and mononuclear macrophages ↓
GM-CSF receptor antagonists	Axatilimab	granulocytes and mononuclear macrophages ↓
IFN-*γ* antagonists	Emapalumab	IFN-*γ*↓
TNF antagonists	Infliximab Adalimumab Golimumab	TNF↓ TNF↓ TNF↓
***Vaccine***		
Inactivated vaccine	–	Prevents cytokine storms
Adenovirus vector vaccine	–	Prevents cytokine storms
mRNA vaccine	–	Prevents cytokine storms
DNA vaccine	–	Prevents cytokine storms
Recombinant protein vaccine	–	Prevents cytokine storms
Attenuated influenza virus vector vaccine	–	Prevents cytokine storms
***Others***		
Sphingosine-1-phosphate receptor agonists	Siponimod	Cytokines↓
TNF blockers		
TLR4 antagonist	Eritoran	Cytokines↓
Stem cell therapy	–	Cytokines↓
Blood purification	–	Cytokines↓
Exogenous surfactants	–	Cytokines↓
Nafamostat mesylate	–	Prevents cytokine storms
Convalescent plasma therapy	–	Cytokines↓
CytoSorb	–	Cytokines, DAMPs, PAMPs↓
Intravenous immunoglobulin (IVIG)	–	Immune regulation
Traditional Chinese medicine	Lianhua–Qingwen formula	Immune regulation

IL-1, interleukin 1; IL-1β, interleukin 1 beta; IL-6, interleukin 6; IL-8, interleukin 8; IL-10, interleukin 10; TNF, tumor necrosis factor; IFN-γ, interferon gamma; GM-CSF, granulocyte-macrophage colony-stimulating; M-CSF, macrophage colony-stimulating factor; DAMPs, damage-associated molecular patterns; PAMPs, pathogen-associated molecular patterns.

Glucocorticoids, which have a good anti-inflammatory effect, may play a vital role in the treatment of COVID-19. Glucocorticoids with a genomic mechanism inhibit the synthesis of proinflammatory cytokines such as IL-1, IL-2, IL-6, IL-8, TNF, IFN-*γ*, COX-2, VEGF, and prostaglandins. Prednisolone, for example, inhibits the production of TNF, IFN-*γ*, IL-1β, IL-6, IL-17, IL-10, and TGF-*β*. Dexamethasone significantly reduces the level of IL-6. Corticosteroids are known to suppress inflammation by non-genomic mechanisms such as inbinding to the membrane-associated glucocorticoid receptors of T cells resulting in perturbation of receptor signaling and immune response and interacting with the exchange of calcium-sodium across the cell membrane, resulting in a quick downturn in inflammation (53). Although studies have revealed that glucocorticoids do not improve the survival of patients with severe COVID-19 (54), dexamethasone was the first drug shown to reduce mortality in COVID-19 patients (55). Recent studies have shown that elderly patients with severe COVID-19 who received systemic corticosteroid therapy have lower in-hospital mortality than patients who did not receive corticosteroid therapy. The study also demonstrated that it is a safe treatment with few serious adverse events (56). This indicates that glucocorticoids may be used in combination with other intensive treatments that may have significant effects.

Focusing on responses to the cytokine storm in the treatment of COVID-19, trials are currently underway for several cytokine antagonists and cytokine receptor antagonists, including IFN, IL-6, IL-6 receptor, IL-1 receptor, IL-1, GM-CSF, and GM-CSF receptor antagonists (57). A new adjuvant therapy, CytoSorb, reduces the circulating levels of cytokines, DAMPs, and PAMPs, and improves immunopathology by absorbing a wide range of these factors (58).

Immunomodulators such as thalidomide that were reported to be effective in treating one COVID-19 patient (59) can inhibit TNF, IL-1, IL-6, and other cytokines (60). However, this was an isolated case, and its efficacy requires further verification (61). Another immunosuppressant—hydroxychloroquine or chloroquine—has shown apparent efficacy in treatment of COVID-19-associated pneumonia in clinical studies (62), but its efficacy remains controversial. A combination of azithromycin and hydroxychloroquine has been reported to yield better results, especially in the early stages of the disease (63). Studies have shown that statin use is associated with a significant reduction in mortality among hospitalized COVID-19 patients (64). Statins can be effective in treating COVID-19 patients as they inhibit the MYD88/NF-*κ*B proinflammatory pathway and promote the upregulation of ACE2 (65).

Based on the pathophysiology of SARS-CoV-2 and the life cycle of the virus, several antiviral drugs will serve as effective anti-COVID-19 targets. Drugs such as remdesivir, favipiravir, and lopinavir/ritonavir have been shown to be effective (66). However, at present, most of these therapeutic drugs are still under clinical trials, and their efficacy against SARS-CoV-2 has not been determined holistically.

Additional treatments that inhibit inflammatory responses, such as TNF blockers, ulinastatin, sphingosine-1-phosphate receptor agonists, Toll-like receptor 4 (TLR4) antagonists, stem cell therapy (especially mesenchymal stem cells) (67), exogenous surfactants (68), intravenous immunoglobulin (IVIG), Nafamostat mesylate (69), convalescent plasma therapy (70), and blood purification therapy, also remain to be further explored and have the potential to effectively treat patients with COVID-19. Traditional Chinese medicine has also elicited positive effects in the treatment of COVID-19. For instance, the Lianhua–Qingwen formula can alleviate symptoms of COVID-19 by activating key molecules such as antiviral and anti-inflammatory synergids (71).

Vaccines are the most reliable and cost-effective approach to avoid and manage infectious diseases. They are designed to build an appropriate and effective immune response without creating any imbalance. There are a number of vaccines in the research phase, including inactivated, live attenuated, viral vector-based, subunit, DNA, and RNA vaccines. In phase I clinical trials of the recombinant adenovirus type-5 vectored COVID-19 vaccine, all 108 volunteers showed significant cellular immune responses (72). mRNA vaccines have rapidly advanced into clinical trials, but most candidate DNA vaccines are currently in the preclinical stage. Moreover, the mRNA-1273 vaccine induced an immune response against SARS-COV-2 in all participants and was relatively safe (73). Now, the mRNA-1273 vaccine is currently in the phase 3 clinical trial. Another mRNA vaccine, the BNT162 (BioNTech, Mainz, Germany), is in advanced clinical trials and has four variants, namely, a1, b1, b2, and c2 (74). Currently, mutations in the virus pose a challenge in vaccine development and production. In a phase II study in South Africa, AstraZeneca/Oxford adenovirus vector vaccine (Chadox1) was only 10.4% effective against mild-to-moderate infection caused by B.1.351 (75). The development and application of vaccines are still met with many challenges, and future challenges are also anticipated.

## Conclusion

The pathogenesis of COVID-19 involves multiple cytokine pathways. Among them, the cytokine storm caused by SARS-CoV-2 is the key reason for deterioration within a short time-span in COVID-19 patients and subsequent development into acute respiratory distress syndrome (ARDS), leading to respiratory failure and eventual death from multiple organ failure. Increasing evidence suggests that cytokines such as IL-6, IL-1β receptor, IFN-*γ*, and TNF-α play a key role in the pathogenesis of COVID-19. Therefore, it is of great significance to accurately identify COVID-19 inflammatory pathways and therapeutic targets. Although many current therapies are based on inhibiting the occurrence and development of cytokine storms, the efficacy of the current therapies is still unsatisfactory, and more research is needed to unlock more key inflammatory pathways triggered by SARS-CoV-2.

## Author Contributions

PZ and YG conceived and designed the study. RC and ZL performed the literature search and drafted the manuscript. All authors contributed to the article and approved the submitted version.

## Funding

Guangdong Medical Science and Technology Research Foundation (A2020162, C2019119, B2021182), Higher Education Teaching Reform Project of Southern Medical University (JG2019126, 2018JG43) Scientific Research Project of Chaozhou Health Bureau (2019110).

## Conflict of Interest

The authors declare that the research was conducted in the absence of any commercial or financial relationships that could be construed as a potential conflict of interest.
